# Completing bacterial genome assemblies: strategy and performance comparisons

**DOI:** 10.1038/srep08747

**Published:** 2015-03-04

**Authors:** Yu-Chieh Liao, Shu-Hung Lin, Hsin-Hung Lin

**Affiliations:** 1 Institute of Population Health Sciences, National Health Research Institutes, Miaoli 350, Taiwan

## Abstract

Determining the genomic sequences of microorganisms is the basis and prerequisite for understanding their biology and functional characterization. While the advent of low-cost, extremely high-throughput second-generation sequencing technologies and the parallel development of assembly algorithms have generated rapid and cost-effective genome assemblies, such assemblies are often unfinished, fragmented draft genomes as a result of short read lengths and long repeats present in multiple copies. Third-generation, PacBio sequencing technologies circumvented this problem by greatly increasing read length. Hybrid approaches including ALLPATHS-LG, PacBio corrected reads pipeline, SPAdes, and SSPACE-LongRead, and non-hybrid approaches—hierarchical genome-assembly process (HGAP) and PacBio corrected reads pipeline via self-correction—have therefore been proposed to utilize the PacBio long reads that can span many thousands of bases to facilitate the assembly of complete microbial genomes. However, standardized procedures that aim at evaluating and comparing these approaches are currently insufficient. To address the issue, we herein provide a comprehensive comparison by collecting datasets for the comparative assessment on the above-mentioned five assemblers. In addition to offering explicit and beneficial recommendations to practitioners, this study aims to aid in the design of a paradigm positioned to complete bacterial genome assembly.

The advent of second-generation sequencing technology has changed the traditional Sanger sequencing paradigm. The decrease in expense associated with sequencing genomic data has made this technology broadly accessible, and now decentralized laboratories can afford to execute their own internal genome sequencing projects^1^,^2^. However, owing to genome complexity, even for microbial genome reconstruction, it remains challenging to generate complete genome assemblies using second-generation sequencing data^3^,^4^. Pacific Biosciences (PacBio) has developed a process enabling single molecule real time (SMRT) sequencing^5^ to produce a significantly longer read length than that of "second-generation" technologies. The data produced from the so-called "third-generation" PacBio sequencing platform is therefore expected to resolve complex repeats. However, due to intrinsic shortcomings in PacBio reads, *i.e.* low accuracy (~15% error rate), several approaches were proposed to combine the advantages of short and long reads for the completion of microbial genomes. These hybrid approaches including ALLPATHS-LG^6^, PacBio corrected reads (PBcR) pipeline^7^,^8^, SPAdes^9^ and SSPACE-LongRead^10^. In contrast to hybrid approaches, due to the random nature of errors in PacBio long reads, non-hybrid approaches exclusively using long reads to produce *de novo* microbial genome assemblies were developed^4^,^11^.

In 2012, Ribeiro *et al.* published work using ALLPATHS-LG to complete bacterial genomes^6^. The authors proposed a unique methodology and incorporated into ALLPATHS-LG to use Illumina paired-end reads of two libraries—one with short and overlapped fragments and another with long jumps—in addition to PacBio long reads. The ALLPATHS-LG algorithm first merged read pairs from short fragments into single "super-reads" for unipath graph generation, then leveraged the "jumping" reads to fill the voids in the unipath graph. PacBio reads were subsequently incorporated to form consensus reads and patch gaps. Though ALLPATHS-LG was able to generate assemblies without supplying PacBio reads as input, both Illumina paired-end reads, fragments and jumps, were necessary to ensure successful genome assembly. Ribeiro *et al.* has demonstrated that ALLPATHS-LG was able to generate nearly perfect bacterial assemblies; nevertheless, few bacterial genome assemblies were completed using this approach^12^,^13^, to the best of our knowledge. We therefore evaluated the performance of ALLPATHS-LG on the genomic data for which the reference genome was available to provide the practical guidance.

Koren *et al.* proposed a hybrid approach to utilize short, high-fidelity reads to reduce error rates in single-molecule long sequencing reads; they increased the accuracy of long reads from 80% to higher than 99.9% and the corrected sequences were then *de novo* assembled^7^. Such a hybrid approach was called "PBcR pipeline"^14^. Meanwhile, Bashier *et al.* provided a hybrid assembler (AHA) to scaffold contigs from an assembly of second-generation sequence data using PacBio long reads. The authors reconciled the long reads with the short reads to either correct errors or fill gaps in the AHA scaffolds. This hybrid assembly analysis was demonstrated in completing bacterial genomes^8^. SSPACE-LongRead was recently proposed by Boetzer and Pirovano for scaffolding draft assemblies using PacBio long reads. The authors validated SSPACE-LongRead to be better capable of producing nearly complete bacterial genomes than AHA^10^. Additionally, recent upgrades of SPAdes added support for taking short and long reads as inputs in SPAdes 3.0^9^,^14^, allowing hybrid assembly. The aim of this study is to compare the hybrid approaches, including ALLPATHS-LG, PBcR pipeline, SPAdes and SSPACE-LongRead, to bacterial genome completion. However, these hybrid approaches require the preparation of at least two different sequencing libraries. A more efficient strategy entails the development of a simple workflow requiring only one library and sequencing method; non-hybrid approaches, in this vein, have been proposed.

Non-hybrid approaches including hierarchical genome-assembly process (HGAP) and PBcR pipeline via self-correction (abbreviated to "PBcR pipeline(S)" hereafter) were developed to use a single, long-insert shotgun DNA library in conjunction with a PacBio single-molecule, real-time sequencing platform for completing microbial genome assemblies^4^,^11^. In contrast to hybrid approaches, HGAP and PBcR pipeline(S) do not require highly accurate short reads for error correction yet require 80-100X of PacBio sequence coverage for self-correction^14^. The key component of HGAP is to develop a consensus algorithm which exploits the inherent advantages of SMRT sequencing quality values to preassemble long and highly accurate overlapping sequences by correcting errors on the longest reads using shorter reads from the same library^11^. The authors who proposed the PBcR pipeline (Koren *et al.*^7^) improved the correction algorithm to perform self-alignment and correction, and released the version of error correction algorithm at http://www.cbcb.umd.edu/software/PBcR/closure/4. The PBcR pipeline is therefore also capable of performing self-correction and non-hybrid assembly for exclusive PacBio reads, we refer to this non-hybrid approach as PBcR pipeline(S). The HGAP software is, in fact, a derivative of PBcR pipeline and is implemented in SMRT Analysis 2.0 or higher. Single molecule sequencing data provided in these two publications were downloaded and analyzed by both non-hybrid approaches separately.

This article reviews strategy and provides a performance comparison among the various methods to complete bacterial genome assemblies. While the algorithms were documented in individual research papers, each work used different datasets for evaluation, rendering cross-comparisons difficult. We thus collected datasets for the comparative assessment on the above-mentioned assembly approaches. We used QUAST^15^ along with the NCBI reference sequences to assess the quality of assemblies generated by the various approaches. We also generated assembly dot plots for the sake of comparison against the reference genome to evaluate the assembly's accuracy by r2cat^16^. We aim to highlight the experimental design on library preparation for each method and provide explicit guidance for practitioners. The detailed procedures, along with the analyzed data and thoroughly-evaluated results, are available online (http://sb.nhri.org.tw/comps).

## Methods

### Assemblers

ALLPATHS-LG is fully automated and requires minimal operator intervention. Prior to executing ALLPATHS-LG, we prepared the data for import into the pipeline; we gathered the read data in the appropriate formats and subsequently provided two information files, including in_groups.csv and in_libs.csv to perform ALLPATHS-LG (release 44837)^6^. SPAdes 3.1 was used for assembly of short reads and hybrid assembly of short and long reads^17^. SPACE-LongRead 1.1 was used to scaffold the SPAdes-assembled contigs from short reads using PacBio long reads^10^. The pre-compiled source code executed under PBcR pipeline was downloaded from http://www.cbcb.umd.edu/software/PBcR/closure/; which is identical to the PBcR pipeline(S)^4^. The latest PBcR pipeline implemented in Celera Assembler 8.2 (wgs-8.2) was also downloaded and used for hybrid and non-hybrid assembly^18^. The HGAP executive programs are implemented in SMRT Analysis 2.0 or higher. We downloaded and installed SMRT v2.0.1 in order to implement HGAP and Quiver^11^. PacBio produces data in HDF5 format (*.h5); the corresponding input file of SMRT Analysis is a bas.h5 or an associated bax.h5 file. All the other assemblers including ALLPATHS-LG, PBcR pipeline, SPAdes, and SSPACE-LongRead expect filtered subreads in fasta or fastq format as an input file. We executed SMRT Analysis to produce subreads by trimming and filtering the raw reads with the following parameters: minSubReadLength = 50, readScore = 0.75 and minLength = 50. We used QUAST 2.3^15^ and r2cat^16^ to evaluate assemblies. We performed all analysis on a server with Intel Xeon E7-4820 processors 8-core 2.00 GHz and 256 GB of RAM.

### Data

To evaluate the assemblers on bacterial genome completion, we collected available sequencing data mainly from the three studies, ALLPATHS-LG, PBcR pipeline(S) and HGAP^4^,^6^,^11^, based on the existence of reference genomes. Because PacBio RS machine was upgraded to PacBio RS II, we also downloaded the data from a single SMRT cell produced by the latest system. The nine different datasets of the five bacterial species employed in this study are summarized and the brief descriptions of libraries are provided in Table 1. For examples, with respect to the dataset 1 of *E. coli*, we downloaded the three-library sequencing data and used NC_000913 as reference genome. Because *R. sphaeroides* 2.4.1 has two chromosomes and five plasmids, the seven reference sequences including NC_007488, NC_007489, NC_007490, NC_007493, NC_007494, NC_009007, and NC_009008 were used for assembly evaluation. Please note that according to the definition of microbial genome complexity described in Koren *et al*'s publication^4^, *M. ruber* DSM 1279 belongs to Class III genome (a maximum repeat size is greater than 7 Kbp) while the other four species are class I genomes (have few repeats other than the rDNA operon sized 5–7 Kbp). The first type of hybrid approach designed for ALLPATHS-LG is to combine two short libraries (short overlapping and jumping reads) with one long library (Table 1, D1–D3). Another type of hybrid approach is to combine one short library with one long library (D4 and D5 in Table 1). In contrast to the hybrid approaches, the non-hybrid approach requires single-library long reads. We therefore employed the five datasets (D5–D9, shown in Table 1) including three species, *E. coli*, *M. ruber* and *R. heparinus*, for non-hybrid assembly evaluation.

## Results and disscusion

### Five assemblers including ALLPATHS-LG, SPAdes, SSPACE-LongRead, PBcR pipeline and HGAP were used and compared in this study

**Figure 1:**
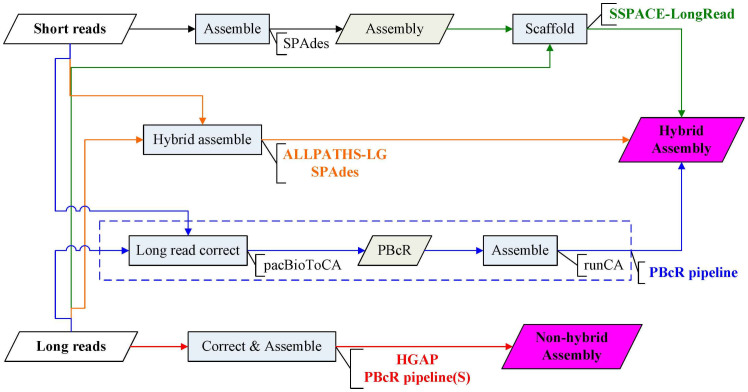
Comparisons of the assemblers conducted in this study. SSPACE-LongRead is a scaffolder using single molecule long reads to upgrade pre-assembled contigs constructed from short reads. ALLPATHS-LG and SPAdes are hybrid assemblers which take short reads and long reads as inputs. PBcR pipeline uses short reads to correct long reads by pacBioToCA, and then assembles corrected long reads (PBcR) by Celera assembler (runCA). Hierarchical genome-assembly process (HGAP) and PBcR pipeline via self-correction (PBcR pipeline(S)) take long reads as input to produce non-hybrid assembly.

As compared and evaluated in GAGE-B, a single library of short reads could not be completely *de novo* assembled by various assemblers into finished genomes, and a jumping library was still necessary to produce large scaffolds^19^. Besides, two recent publications have demonstrated that hybrid assemblies combining 454 with two paired Illumina libraries (fragment reads and jumping reads) did not produce complete genomes^14^,^20^. In order to provide a strategy for bacterial genome completion, we have surveyed the assemblers that are able to utilize the PacBio long reads. As illustrated by Figure 1, ALLPATHS-LG and SPAdes are the two hybrid assemblers that take short and long reads as inputs to perform *de novo* assembly^6^,^14^. SSPACE-LongRead is designed to scaffold pre-assembled contigs using long reads^10^. PBcR pipeline uses short reads to correct long reads and then to *de novo* assemble the corrected PacBio long reads (PBcR)^4^,^7^,^14^. In addition to the hybrid approaches, non-hybrid approaches—HGAP^11^ and PBcR pipeline(S)^4^—were used in this study. As summarized in Table 1, the nine datasets were used to evaluate the five assemblers on bacterial genome completion. Ribeiro *et al.* has employed ALLPATHS-LG to assemble 16 bacterial samples and hence has generated nearly perfect genome assemblies in some cases^6^. In order to evaluate the performance of ALLPATHS-LG on reproducing the bacterial genome assemblies, we have executed the routine on the sequence data (Table 1, D1–D3). Some of the identical datasets were hybrid assembled by using SPAdes. As for another type of hybrid approach: to combine one short library with one long library, we used PBcR pipeline, SPAdes and SSPACE-LongRead to assemble the reads from Dataset 4 and Dataset 5. Besides, HGAP and PBcR pipeline(S) were conducted for non-hybrid assemblies on the long reads of the three species (Table 1, D5–D9)

### ALLPATHS-LG completed bacterial genomes under a well-controlled coverage

In addition, to leverage ALLPATHS-LG on the datasets of reads available on Ribeiro's ftp (see Table 1, D1–D3), the raw short reads were directly downloaded from the Sequence Read Archive (SRA). The results of the assembly operation can be found in Table 2, in terms of number of contigs and N50; the results generated from the website data strongly corroborate the results obtained in the previous study^6^. The details of assemblies evaluated by QUAST are shown in Additional file 1: Table S1–S3. Single contigs were generated for *E. coli* and *S. pneumoniae* while 11 contigs were generated for *R. sphaeroides*. Furthermore, the assembly results exhibited parallelism to the reproducible results obtained from the website data, which occurred when the fraction of reads in ALLPATHS-LG was specified to be identical to the website data, *i.e.* the fractions of fragment reads and jumping reads were set to 0.088 and 1 (for *E. coli*), 0.384 and 1 (for *R. sphaeroides*), and 0.187 and 1 (for *S. pneumoniae*), respectively. These fractions of read data are equivalent to approximately 52X, 191X and 100X genome coverage for fragment libraries and 79X, 87X, 100X genome coverage for jump libraries of the three aforementioned species. However, the assemblies appeared to manifest less accurate results when the raw data was utilized in ALLPATHS-LG (over 497X genome coverage in the fragment libraries), which suggests that the effect of coverage on the assembly methodology must be explored in further detail. In this vein, ALLPATHS-LG was firstly performed on the data with 50X genome coverage according to the laboratory formula described in its publication^6^. ALLPATHS-LG, in the case of *E. coli*, generated a single contig exhibiting nearly perfect accuracy. The approach assembled two contigs for *S. pneumoniae* but was unable to produce an assembly for *R. sphaeroides* at as low as 50X coverage. As discussed by Ribeiro *et al.*, coverage is difficult to control due to sample-to-sample variability; ALLPATHS-LG was employed to process the 100X genome coverage data to ensure that steady assemblies could be obtained. As is evident from the results (Table 2 and Additional file 2: Figure S1–S3), a customized implementation of ALLPATHS-LG is able to complete accurate bacterial genomes.

### ALLPATHS-LG produced accurate but gapped assemblies in the absence of long reads

ALLPATHS-LG has been proposed to complete bacterial genomes in which it explicitly requires minimum of two libraries (short and jumping libraries)^6^. However, to the best of our knowledge, few bacterial genomes have been completed using this strategy^12^,^21^. It is therefore speculated that the methodology that concatenates three data types generated from Illumina and Pacific Biosciences impedes the applicability of ALLPATHS-LG. Ribeiro *et al.* has examined the use of ALLPATHS-LG closely, the authors have not supplied the algorithm with long reads to evaluate its performance limits and have stated that "the omission of long reads cuts at the heart of the method and would be expected to have deleterious effects". In this and other cases, ALLPATHS-LG is often used without long reads^13^,^21^,^22^. To this end, we assessed the performances of ALLPATHS-LG without supplying long reads (Table 2). Although ALLPATHS-LG could produce nearly complete genome assemblies for *E. coli*, the number of uncall bases (N's), representing gaps in the scaffolds, substantially increase in the absence of long reads (e.g., from 0 to 533 per 100 Kbp in the assemblies obtained from Website data), which corresponds to the role of long reads in filling gaps^6^ (see Additional file 1: Table S1–S3). As per the effect of coverage, similar results can be found in Table 2 (comparing with and without PacBio), *i.e.* extremely high coverage is not necessary for optimal assembly. Evidently, ALLPATHS-LG was impeded from the generation of complete genomes by the lack of long reads; although diagonal-like dot plots against reference genomes (Addition file 2: Figure S4–S6) were observed, the accurate assemblies were gapped and sometimes fragmented.

### SPAdes did not fully utilize the data designed for ALLPATHS-LG. It assembled short reads with long reads efficiently

We applied SPAdes to hybrid assemble the datasets originally designed for ALLPATHS-LG (Table 1, D1–D3), but got unsatisfied assemblies (Table 2). Dozens of contigs were generated even if the PacBio long reads were used, and the N50 values obtained from SPAdes were as low as one tenth of the values obtained from ALLPATHS-LG. We speculated that the requirement of ALLPATHS-LG—the short fragment library whose insert lengths are slightly shorter than twice the read lengths—is not optimal to SPAdes. Additionally, the PacBio long reads used in ALLPATHS-LG are 1 ~ 3 Kbp, such a length may not long enough for SPAdes to perform efficient hybrid assembly. We have replaced the long reads of the Dataset 1 with the data of a single-SMRT cell from the Dataset 5, and the assembly result obtained from SPAdes was obviously improved (N50 from 1 Mbp to 3 Mbp, Additional file 1, Table S1), which suggests that 10 Kbp long read library is benefit to SPAdes for producing high quality assemblies. Although SPAdes was unable to produce a single-contig assembly with additional long reads from a single SMRT cell, it generated the highest N50 statistics in comparison with the results from PBcR pipeline and SSPACE-LongRead using a small amount of long reads (one and two SMRT cells in Table 3). In addition, SPAdes generated assemblies within 3 hours. Moreover, SPAdes was capable of reconstructing the genome of *E. coli* as the latest PacBio RS II long reads (Dataset 9) were hybrid assembled with the short reads (Dataset 4) (see Additional file 1: Table S4 and Additional file 2: Figure S7 for details).

### Hybrid assembly from one short and one long library was inefficient to complete bacterial genome

To assemble the hybrid data (one short and one long library), we conducted PBcR pipeline, SPAdes and SSPACE-LongRead on the dataset D4 + D5. We found that several factors influence assembly results generated by PBcR pipeline, such as read depth, specifying genome size or not, and Celera Assembler parameters. The detailed descriptions are provided in Additional file 2: Supporting data. In short, the expected genome size should be specified in long read correction (pacBioToCA), the 25X longest PBcR should be used for assembly (runCA), and the contigs with fewer than 100 mapped PacBio corrected reads should be discarded. We have followed the procedure of PBcR pipeline carefully; nevertheless, we did not produce single-contig assemblies, even when a substantial body of long reads from the 17 SMRT cells were used (evidence for this is in Table 3). The latest PBcR pipeline was recently released in Celera Assembler wgs-8.2, we thus used it to hybrid assemble Datasets 4 and 5. Unlike the PBcR pipeline available at cbcb, the latest PBcR pipeline provides a single command (PBcR) to perform long read correction and assembly. Albeit the updated PBcR pipeline reduced its running time and increased the N50 statistics, it was unable to produce a single-contig assembly wherein even four SMRT-cell long reads (from D5) were used along with the Dataset 4. The four SMRT cell reads were successfully non-hybrid assembled into a single-contig using the identical pipeline (PBcR pipeline(S) in wgs-8.2). Because SSPACE-LongRead required pre-assembled contigs, we used SPAdes to assemble the Illumina short reads of the Dataset 4, then scaffolded the assembly with long read data from one to four SMRT cells and from 17 SMRT cells of the Dataset 5. The QUAST-evaluated results are shown in Table 3 (see Additional file 1: Table S4 for details). With the addition of long reads from a single-SMRT cell, the assembly N50 was increased from 139 Kbp to 2.4 Mbp using SPAdes or SSPACE-LongRead, which shows that the utilization of PacBio long reads is great capable of upgrading draft assembly constructed from short reads. Besides, in terms of running time, SPAdes and SSPACE-LongRead produced the hybrid assembles in a couple of hours. As described in the previous paragraph, SPAdes reconstructed the genome of *E. coli*, with the largest contig over 4.6 Mbp, when either the PacBio RS II data (D9) or the 17 SMRT cell data (D5) was used to hybrid with the short reads (D4) (as shown in Table 3). Nevertheless, with the given data, SSPACE-LongRead did not scaffold the SPAdes-assembled contigs into a single contig (see Additional file 1: Table S4 and Additional file 2: Figure S7–S8 for more details). Several studies investigated the effect of coverage on genome assemblies and found that the N50 length plateau was reached at 75X of coverage^23^. We therefore sub-sampled 75X of short reads from Dataset 4 to hybrid assemble with long reads from Dataset 5 using SPAdes. While the N50 lengths (compared to Table 3) were increased from 1.2 Mbp and 1.7 Mbp to 2.5 Mbp and 3.8 Mbp, respectively, in which three and four SMRT cell long reads were used, SPAdes was not able to complete *E. coli*'s genome. Taken together, incorporating long reads (15X-40X) with short reads was promising to enhance the continuity of incomplete draft assemblies constructed from short reads by using SPAdes; however, such a hybrid approach was inefficient in producing complete bacterial genomes.

### Non-hybrid approaches required as few as one single PacBio RS II SMRT cell to complete bacterial genome

With SMRT Analysis v2.0.1, we were able to conduct HGAP procedure and Quiver algorithm for bacterial non-hybrid *de novo* genome assembly^11^. Similar to HGAP, the PBcR pipeline is also capable of performing self-correction and non-hybrid assembly of PacBio reads when sufficient coverage is available^4^,^14^. Although Koren *et al.* has recommended 150X sequencing depth to facilitate the completion of an accurate microbial genome^4^, Chin *et al.* has demonstrated that as few as three SMRT cells (RS I system, equivalent to 90X) are sufficient to produce a single contig^11^. As was apparent from the work, read length and depth determine assembly continuity; we therefore conducted HGAP and PBcR pipeline(S) on various SMRT cells, ranging from 4 to 17 XL-C2 SMRT cells generated from the PacBio RS I system (Table 1, D5–D8), and on a single SMRT cell gathered with PacBio RS II system and P4–C2 chemistry (Table 1, D9). The detailed procedures and QUAST-evaluated assembly results (see Additional file 1: Table S5–S6) are provided on our website. As we can see from Table 4, HGAP or PBcR pipeline(S) is capable of producing single contigs except for the dataset D7, which embodies a sequencing coverage of 124X. Nevertheless, *Chin et al.* has generated a single contig from three SMRT cells of the dataset D7 and stated that the assembly from the four SMRT cells contained one misassembly with respect to the reference genome^11^; The misassemblies are highlighted with underline as listed in Table 4 and the dot plots of sequence assemblies against the reference genome are displayed in Additional file 2: Figure S9–S10. Interestingly, employing more sequencing reads does not always guarantee the perfect assembly. For example, 4 SMRT cells (70X ~ 77X) of dataset D5 were sufficient to produce a single contig but several of the 6 and 8 SMRT cells did not result in perfect assemblies. Similarly, PBcR pipeline(S) successfully assembled the genome of *E. coli* into a single contig using 6 SMRT-cell reads, but misassembled two contigs (the large contig was unable to correctly align on the reference genome) when applying the 8 SMRT-cell data of dataset D6 (Additional file 2: Figure S10). It is therefore recommended to execute a small number of SMRT cells produced from PacBio RS I system (e.g. 3 or 4). Additional SMRT cells can be gradually appended, if necessary. Moreover, current upgrades (PacBio RS II) increase the throughput and read length yielded from a single SMRT cell. Those sequencing reads (Dataset 9) were successfully assembled by both HGAP and PBcR pipeline(S) into a single contig, as indicated in Table 4. Recently, a complete genome of *C. autoethanogenum* DSM10061 has been published by performing HGAP to analyze single molecule reads produced by PacBio RS II without the need for manual finishing^20^, which supports the assertion that the PacBio single-molecule technology will be valuable in future studies. Although the runtime of HGAP and PBcR pipeline(S) on the Dataset 9 was 16 and 31 hours, respectively (Additional file 1: Table S5), it only took 24 minutes and 2.3 hours using the latest version of PBcR pipeline (wgs-8.2) and HGAP 3.0 (under SMRT v2.3.0) to reconstruct the genome of *E. coli* from the identical dataset (D9) (Table S7 and S9). Both non-hybrid approaches are capable of completing microbial genomes; nevertheless, they each seem to possess unique advantages in finishing various bacterial genomes. As a whole, we suggested that practitioners should perform both non-hybrid approaches to *de novo* assemble a bacterial genome, or at least should ask a PacBio sequencing provider to run HGAP.

### The latest version of non-hybrid approaches rapidly produced accurate and complete bacterial genome

As illustrated in the previous paragraph, the running time for the non-hybrid approaches was greatly reduced (from over 10 hours to 30 minutes). The latest Celera Assembler wgs-8.2 including PBcR pipeline currently incorporates a novel probabilistic overlapper (named MHAP) for self-correction^24^. Such the implementation speeds up the assembly process. While we have found that the genome size should be specified for hybrid assembly (Additional file 2), the effects of genome size setting on non-hybrid approach remained unclear. We performed the varied non-hybrid assemblies with the expected genome size using PBcR pipeline(S) at cbcb and HGAP 2.0, the results are shown in Table 4. However, an exact genome size was mostly unavailable for an undetermined bacterial genome. We therefore conducted the Celera Assembler wgs-8.2 with different genome size settings (without genome size and the ratio to the genome size from 0.8 to 1.2) on the dataset D5–D9 to examine the completeness and accuracy of assembly production. Note that we discarded the contigs with fewer than 25 mapped long reads, from assemblies obtained by wgs-8.2. As can be seen in Table 5, while the coverage of long read is crucial to produce a complete genome, the parameter of genome size setting is no longer an issue (see Additional file 1: Table S7 and S8 for the detailed QUAST-evaluated results). The latest PBcR pipeline in wgs-8.2 was able to generate a single-contig assembly, even for the Class III *M. ruber* (max. repeat size >7 Kbp), as the coverage was over 75X, except when applied to Dataset 6. We ascribed this exception to a higher coverage bias (given with a couple identified low-coverage regions, details are available at our website). Besides, the latest PBcR pipeline produced accurate assemblies in connection to no large structure error (evaluated by r2cat) while it was unable to resolve large repeats in *P. heparinus* (>5 Kbp) and led to misassemblies (Additional file 2: Figure S11). As PBcR supported two alternate consensus modules, we performed the faster algorithm by specifying -pbCNS on the data, the assemble results are summarized in Table 5. In order to resolve the misassmblies in *P. heparinus*, we used the default consensus module PBDAGCON to gain the accurate assemblies except one. However, a double running time was required for this consensus module (from 30 min to 1 hour). Nevertheless, the upgraded RS II system increased the average read length to 5 Kbp (in Dataset 9) and expectedly provided average read lengths in excess of 10 Kbp with new chemistry (P6-C4), which allows the full closure of most bacterial genome. Because we were unable to successfully load the old HDF5 format, generated from RS I system, into the latest HGAP 3.0, we run the HGAP 3.0 on the Dataset 9 with different setting of genome size. Similar to the results obtained from the latest PBcR pipeline (wgs-8.2), HGAP 3.0 produced accurate single-contig assemblies without the interference of genome size setting, however, it took more than 2 hours to generate an assembly (Additional file 1: Table S9). Note that the assemblies were polished by Quiver in HGAP 3.0 to provide highly consensus accuracy. The running time was correspondingly increased. We polished the assembly produced by the latest PBcR pipeline on D9, the consensus accuracy was improved from 99.96 to 99.9997% with an extra running hour. Taken together, the latest algorithms implemented in the non-hybrid approaches successfully produced accurate and complete bacterial genomes in a reasonable time.

## Conclusions

With the advent of technologies in PacBio single-molecular real-time sequencing, the read length and the throughput are continuously increased. The non-hybrid approach relying on single-library preparation is the preferred way to *de novo* assemble and thereby complete bacterial genomes. Although it took us more than a day to perform a non-hybrid bacterial genome assembly using both HGAP 2.0 and the PBcR pipeline(S) at cbcb, the latest version of Celera Assembler (wgs-8.2, including PBcR pipeline) was capable of producing the bacterial genome assembly within 30 minutes. To the point of view on the inefficiency of hybrid assemblies (in terms of multiple library preparation and the capability of producing single contigs), we therefore recommended the practitioners to exclusively sequence their bacterial genomes using PacBio RS II system. In cooperation of the latest non-hybrid approaches, bacterial genomes can be efficiently reconstructed by either PBcR pipeline(S) in wgs-8.2 or HGAP 3.0. It is anticipated that future technological advancements in PacBio chemistry and technology will further extend the reach of microbial genome assembly—the known microbial genome to be sequenced and completed.

## Author Contributions

Y.C.L. conceived and designed the study. H.H.L. and S.H.L. conducted all analyses. S.H.L. and Y.C.L. wrote the manuscript. All the authors read and approved the final manuscript.

## Supplementary Material

Supplementary Information Additional file 1

Supplementary Information Additional file 2

## Figures and Tables

**Table 1:** Description of the datasets employed in this study

Data	Organism	Fragment	Jump	Long read	Reference
D1	*E. coli* K-12 MG1655	2 × 101 bp, 180 bp insert (SRR447685)	2 × 93 bp, 3000 bp insert (SRR401827 and SRR492488)	1–3 Kbp (Ribeiro's ftp^a^)	NC_000913
D2	*R. sphaeroides* 2.4.1	2 × 101 bp, 180 bp insert (SRR125492)	2 × 101 bp, 3000 bp insert (SRR388672)	1–3 Kbp (Ribeiro's ftp^a^)	NC_007488-90 NC_007493-94 NC_009007-08
D3	*S. pneumoniae* TIGR4	2 × 101 bp, 180 bp insert (SRR387335)	2 × 93 bp, 3000 bp insert (SRR364158)	1–3 Kbp (Ribeiro's ftp^a^)	NC_003028
D4	*E. coli* K-12 MG1655	2 × 151 bp, 300 bp insert (Illumina data website^b^)			NC_000913
D5	*E. coli* K-12 MG1655			10 Kbp, 17 SMRT cell (SRX255228^c^)	NC_000913
D6	*E. coli* K-12 MG1655			8–10 Kbp, 8 SMRT cells (SRX260475^d^)	NC_000913
D7	*M. ruber* DSM 1279			8–10 Kbp, 4 SMRT cells (SRX260496^d^)	NC_013946
D8	*P. heparinus* DSM 2366			8–10 Kbp, 7 SMRT cells (SRX260506^d^)	NC_013061
D9	*E. coli* K-12			PacBio RS II System and P4-C2 chemistry^e^, 20 Kbp library, 1 SMRT cell	NC_000913

^a^Long reads were downloaded from ftp.broadinstitute.org/pub/papers/assembly/Ribeiro2012/data.

^b^Paired reads were provided in http://www.illumina.com/systems/miseq/scientific_data.ilmn.

^c^PacBio HDF5 files were requested from NCBI Sequence Read Archive (SRA).

^d^PacBio HDF5 files were downloaded from http://files.pacb.com/software/hgap/index.html.

^e^PacBio HDF5 files were downloaded from https://github.com/PacificBiosciences/DevNet/wiki/E.-coli-20kb-Size-Selected-Library-with-P4-C2.

**Table 2:** Assembly results obtained by ALLPATHS-LG and SPAdes on D1–D3

	ALLPATHS-LG				SPAdes			
	With PacBio		Without PacBio		With PacBio		Without PacBio	
	No. of contigs	N50	No. of contigs	N50	No. of contigs	N50	No. of contigs	N50
*E. coli*	*1 chromosome, genome size: 4639675 bp*							
Website data	1	4638970	2	4631220	16	692096	31	555967
Raw data	1	4625005	1	4633080	28	1092719	40	693826
Fractional data	14	4638970	5	4575759				
50X coverage	1	4638970	3	4629108				
100X coverage	1	4638970	2	4638312				
*R. sphaeroides*	*2 chromosomes and five plasmids, genome size: 4606060 bp, expected N50: 3188609 bp*							
Website data	11	3188818	31	3188995	57	318530	114	183697
Raw data	13	3188540	57	3186675	44	422736	93	223105
Fractional data	10	3188847	32	1492665				
50X coverage	NA	NA	79	99916				
100X coverage	12	3188773	29	2634704				
*S. pneumoniae*	*1 chromosome, genome size: 2160842 bp*							
Website data	1	2162245	4	1663585	20	210016	65	84287
Raw data	5	1340620	6	2135901	90	365564	142	81903
Fractional data	1	2151421	4	1671738				
50X coverage	2	1189234	4	1675149				
100X coverage	1	2150940	7	1812035				

**Table 3:** Assembly results obtained from hybrid one short and one long library (D4 + D5)

SMRT	Hybrid approach	No. contigs	N50	No. misassemblies	No. N's per 100 Kbp	No. genes	Running time
0	SPAdes	86	139882	2	0	4399	2 h 28 m
1	PBcR pipeline	19	356974	8	0.19	4473	Over 12 h
	PBcR pipeline (wgs-8.2)	24	564692	7	0	**4482**	6 h 8 m
	SPAdes	**15**	**2498709**	**6**	**0**	4479	**2 h 34 m**
	SSPACE-LongRead	**15**	2497845	9	97.89	4467	2 h 38 m
2	PBcR pipeline	17	405539	7	**0**	4466	Over 12 h
	PBcR pipeline (wgs-8.2)	**9**	981448	11	**0**	**4495**	9 h 52 m
	SPAdes	14	**3196491**	**6**	**0**	4485	**2 h 36 m**
	SSPACE-LongRead	18	1238868	10	67.67	4465	2 h 47 m
3	PBcR pipeline	15	323732	**6**	**0**	4467	Over 12 h
	PBcR pipeline (wgs-8.2)	**6**	**3385118**	10	**0**	**4495**	10 h 24 m
	SPAdes	12	1241619	**7**	**0**	4492	**2 h 40 m**
	SSPACE-LongRead	16	2501081	10	77.33	4476	2 h 59 m
4	PBcR pipeline	12	834736	9	0.13	4456	Over 12 h
	PBcR pipeline (wgs-8.2)^a^	**2**	**4461262**	9	**0**	**4494**	11 h 57 m
	SPAdes	11	1750947	**7**	**0**	4492	**2 h 35 m**
	SSPACE-LongRead	15	3194637	10	77.37	4477	3 h 3 m
17	PBcR pipeline	5	1215597	8	0.02	4487	Over 12 h
	PBcR pipeline (wgs-8.2)	**3**	**4649343**	11	**0**	**4495**	Over 12 h
	SPAdes	6	4644452	**7**	**0**	**4495**	**2 h 46 m**
	SSPACE-LongRead	17	1238635	8	91.03	4467	5 h 12 m

aIt produced a non-hybrid assembly within 30 min, with a single contig (4.6 Mbp) when using the long reads of 4 SMRT cells.

**Table 4:** Evaluation of the non-hybrid assembly on assembly completeness in terms of the number of contigs; triplicate experiments were performed where applicable

Dataset	Data description	Assembly approach^a^	4 SMRT cells	6 SMRT cells	8 SMRT cells	All SMRT cells
D5	*E. coli*, 17 SMRT cells	HGAP	1, 2, 6	4, 2, 4	2, 2, 3	Fail^b^
		PBcR pipeline(S)	1, 1, 5	1, 2, 2	1, 2, 4	1
D6	*E. coli*, 8 SMRT cells	HGAP	8, 10, 12	4, 9, 12		7
		PBcR pipeline(S)	8, 10, 14	1, 1, 4		2
D7	*M. ruber*, 4 SMRT cells	HGAP				3
		PBcR pipeline(S)				2
D8	*P. heparinus*, 7 SMRT cells	HGAP	2, 2, 5			2
		PBcR pipeline(S)	3, 3, 3			1
D9	*E. coli*, 1 RS II SMRT cell	HGAP				1
		PBcR pipeline(S)				1

^a^HGAP, hierarchical genome-assembly process; PBcR pipeline(S), PacBio corrected reads pipeline via self-correction.

^b^Due to memory limitations, we were unable to load the 17 SMRT cells successfully.

**Table 5:** Evaluation of the latest PBcR pipeline(S) included in wgs-8.2 on assembly completeness and accuracy in terms of the number of contigs and diagonal-like dot plots

Dataset	Genome size setting^a^	3 SMRT	4 SMRT	6 SMRT	8 SMRT	All SMRT
D5 17 SMRT cells	without		|▪	▪▪▪	▪▪▪	▪
	0.8		6□6□|▪	▪▪▪	▪▪▪	▪
	0.9		6□6□|▪	▪▪▪	▪▪▪	▪
	1		4□6□|▪	▪▪▪	▪▪▪	▪
	1.1		5□6□|▪	▪▪▪	▪▪▪	▪
	1.2		4□6□|▪	▪▪▪	▪▪▪	▪
D6 8 SMRT cells	0.8			|7□7□10□		2□
	0.9			|6□7□7□		2□
	1			|6□6□6□		2□
	1.1			|6□6□6□		2□
	1.2			|3□6□6□		4□
D7 4 SMRT cells	without	|▪▪▪▪				
	0.8					▪
	0.9					▪
	1					▪
	1.1					▪
	1.2					▪
D8 7 SMRT cells	without			|▪▪▪		▪
	without^b^			|▪▪▪		▪
	0.8					▪
	0.9					▪
	1					▪
	1.1					▪
	1.2					▪
D9 1 RSII SMRT cell	without					|▪
	0.8					|▪
	0.9					|▪
	1					|▪
	1.1					|▪
	1.2					|▪

▪: Accurate and complete assembly.

: Accurate assembly with diagonal-like dot plot against reference genome, the number in box represents the number of contigs in an assembly.

▪: A single but misassembled contig. The vertical bar represents a cutoff of 75X long reads.

aCommand of PBcR pipeline: PBcR -pbCNS -length 500 -partitions 200 genomeSize = 4650000 (for *E. coli* D5, D6, and D9); genomeSize = 3100000 (for *M. ruber* D7); genomeSize = 5170000 (for *P. heparinus* D8).

bCommand of PBcR pipeline: PBcR -length 500 -partitions 200.

## References

[b1] Finotello F. *et al.* Comparative analysis of algorithms for whole-genome assembly of pyrosequencing data. Briefings in Bioinformatics 10.1093/bib/bbr063 (2011). 22021898

[b2] Quail M. A. *et al.* A tale of three next generation sequencing platforms: comparison of Ion Torrent, Pacific Biosciences and Illumina MiSeq sequencers. BMC Genomics 13, 341, 10.1186/1471-2164-13-341 (2012). 22827831 PMC3431227

[b3] Ferrarini M. *et al.* An evaluation of the PacBio RS platform for sequencing and de novo assembly of a chloroplast genome. BMC Genomics 14, 670, 10.1186/1471-2164-14-670 (2013). 24083400 PMC3853357

[b4] Koren S. *et al.* Reducing assembly complexity of microbial genomes with single-molecule sequencing. Genome Biol 14, R101, 10.1186/gb-2013-14-9-r101 (2013). 24034426 PMC4053942

[b5] Eid J. *et al.* Real-time DNA sequencing from single polymerase molecules. Science 323, 133–138, 10.1126/science.1162986 (2009). 19023044

[b6] Ribeiro F. J. *et al.* Finished bacterial genomes from shotgun sequence data. Genome Res 22, 2270–2277, 10.1101/gr.141515.112 (2012). 22829535 PMC3483556

[b7] Koren S. *et al.* Hybrid error correction and de novo assembly of single-molecule sequencing reads. Nature biotechnology 10.1038/nbt.2280 (2012). PMC3707490 22750884

[b8] Bashir A. *et al.* A hybrid approach for the automated finishing of bacterial genomes. Nature biotechnology 10.1038/nbt.2288 (2012). PMC3731737 22750883

[b9] Prjibelski A. D. *et al.* ExSPAnder: a universal repeat resolver for DNA fragment assembly. Bioinformatics 30, i293–i301, 10.1093/bioinformatics/btu266 (2014). 24931996 PMC4058921

[b10] Boetzer M. & Pirovano W. SSPACE-LongRead: scaffolding bacterial draft genomes using long read sequence information. BMC Bioinformatics 15, 211, 10.1186/1471-2105-15-211 (2014). 24950923 PMC4076250

[b11] Chin C. S. *et al.* Nonhybrid, finished microbial genome assemblies from long-read SMRT sequencing data. Nat Methods 10, 563–569, 10.1038/nmeth.2474 (2013). 23644548

[b12] Shibata T. F. *et al.* Complete Genome Sequence of Burkholderia sp. Strain RPE64, Bacterial Symbiont of the Bean Bug Riptortus pedestris. Genome announcements 1, 10.1128/genomeA.00441-13 (2013). PMC3703598 23833137

[b13] Ku C., Lo W. S., Chen L. L. & Kuo C. H. Complete Genome Sequence of Spiroplasma apis B31T (ATCC 33834), a Bacterium Associated with May Disease of Honeybees (Apis mellifera). Genome announcements 2, 10.1128/genomeA.01151-13 (2014). PMC3886961 24407648

[b14] Utturkar S. M. *et al.* Evaluation and validation of de novo and hybrid assembly techniques to derive high quality genome sequences. Bioinformatics 10.1093/bioinformatics/btu391 (2014). PMC4173024 24930142

[b15] Gurevich A., Saveliev V., Vyahhi N. & Tesler G. QUAST: quality assessment tool for genome assemblies. Bioinformatics 29, 1072–1075, 10.1093/bioinformatics/btt086 (2013). 23422339 PMC3624806

[b16] Husemann P. & Stoye J. r2cat: synteny plots and comparative assembly. Bioinformatics 26, 570–571, 10.1093/bioinformatics/btp690 (2010). 20015948 PMC2820676

[b17] Bankevich A. *et al.* SPAdes: a new genome assembly algorithm and its applications to single-cell sequencing. Journal of computational biology: a journal of computational molecular cell biology 19, 455–477, 10.1089/cmb.2012.0021 (2012). 22506599 PMC3342519

[b18] Myers E. W. A Whole-Genome Assembly of Drosophila. Science 287, 2196–2204, 10.1126/science.287.5461.2196 (2000). 10731133

[b19] Magoc T. *et al.* GAGE-B: An Evaluation of Genome Assemblers for Bacterial Organisms. Bioinformatics 10.1093/bioinformatics/btt273 (2013). PMC3702249 23665771

[b20] Brown S. D. *et al.* Comparison of single-molecule sequencing and hybrid approaches for finishing the genome of Clostridium autoethanogenum and analysis of CRISPR systems in industrial relevant Clostridia. Biotechnology for biofuels 7, 40, 10.1186/1754-6834-7-40 (2014). 24655715 PMC4022347

[b21] Ku C., Lo W. S., Chen L. L. & Kuo C. H. Complete genomes of two dipteran-associated spiroplasmas provided insights into the origin, dynamics, and impacts of viral invasion in spiroplasma. Genome biology and evolution 5, 1151–1164, 10.1093/gbe/evt084 (2013). 23711669 PMC3698928

[b22] Brown S. D. *et al.* Genome Sequences of Industrially Relevant Saccharomyces cerevisiae Strain M3707, Isolated from a Sample of Distillers Yeast and Four Haploid Derivatives. Genome announcements 1, 10.1128/genomeA.00323-13 (2013). PMC3675515 23792743

[b23] Junemann S. *et al.* GABenchToB: A Genome Assembly Benchmark Tuned on Bacteria and Benchtop Sequencers. PLoS One 9, e107014, 10.1371/journal.pone.0107014 (2014). 25198770 PMC4157817

[b24] Berlin K. *et al.* Assembling Large Genomes with Single-Molecule Sequencing and Locality Sensitive Hashing. bioRxiv, 10.1101/008003 (2014). 26006009

