# Supplementary material for: Completing bacterial genome assemblies: strategy and performance comparisons
Source: Sci Rep. 2015 Mar 4;5:8747. doi: 10.1038/srep08747 (PMC4348652; doi:10.1038/srep08747)
Supplement: Supplementary Information — Additional file 2 [file srep08747-s2.pdf]

Additional file 2 and 3

Title:

Completing bacterial genome assemblies: strategy and performance comparisons

Authors:

Yu-Chieh Liao*, Shu-Hung Lin and Hsin-Hung Lin

File name: Additional file 2

Title: Figure S1-S11

Description: Dot plots of sequences assemblies produced by various assemblers

File name: Additional file 3

Title: Supporting data

Description: Detailed descriptions about PBcR pipeline

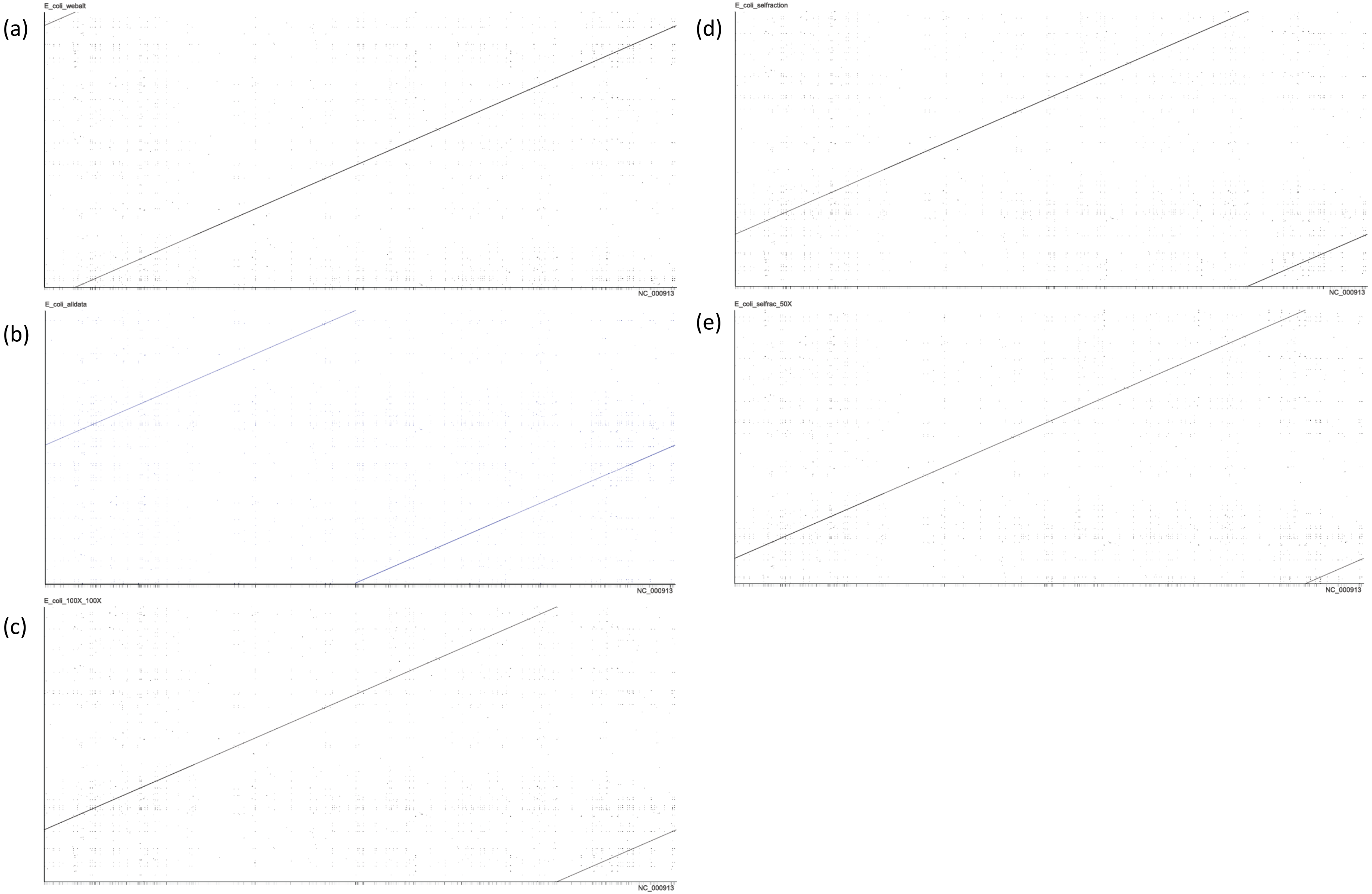

Figure S1 Dot plots of sequences assemblies produced by ALLPATHS-LG on dataset 1. (a) Website data, (b) Raw data, (c) 100X coverage, (d) Fractional data, and (e) 50X coverage.

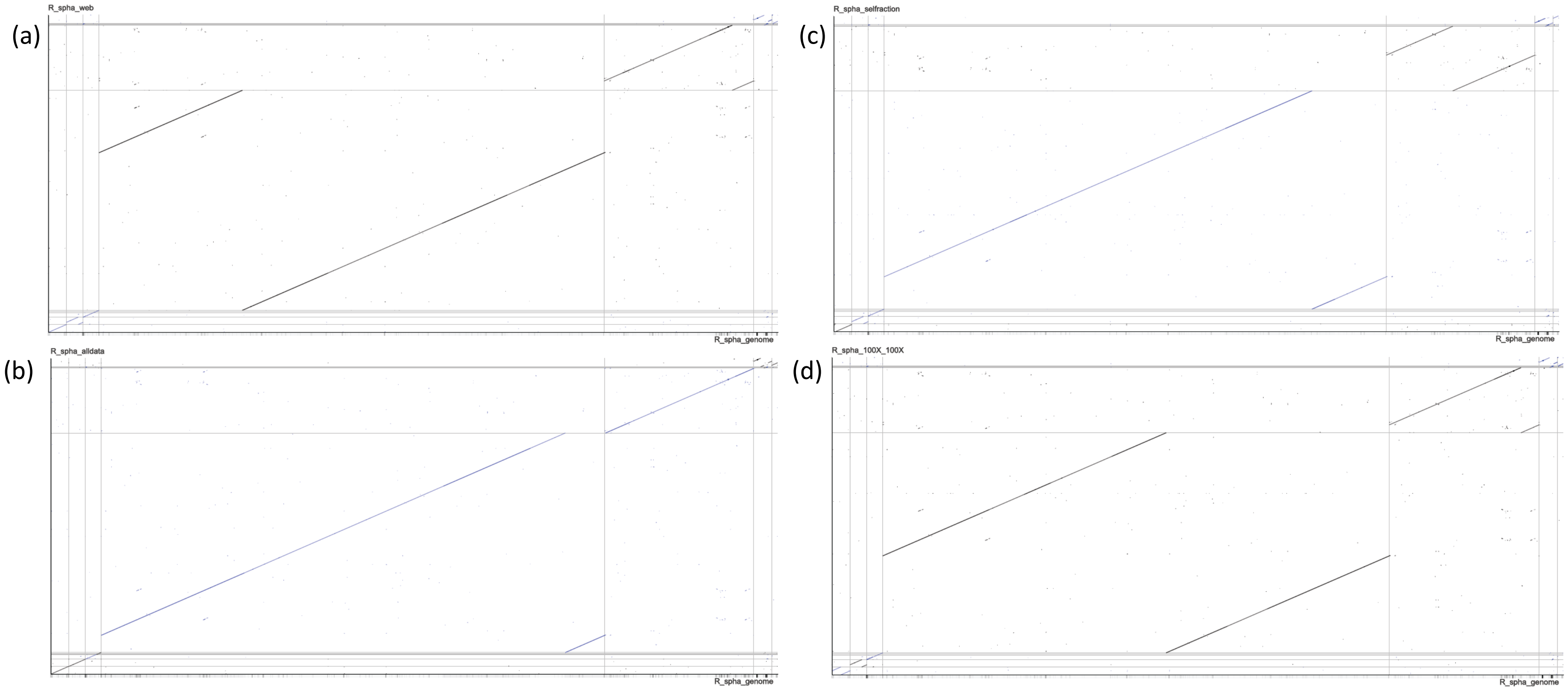

Figure S2 Dot plots of sequences assemblies produced by ALLPATHS-LG on dataset 2. (a) Website data, (b) Raw data, (c) Fractional data, and (d) 100X coverage.

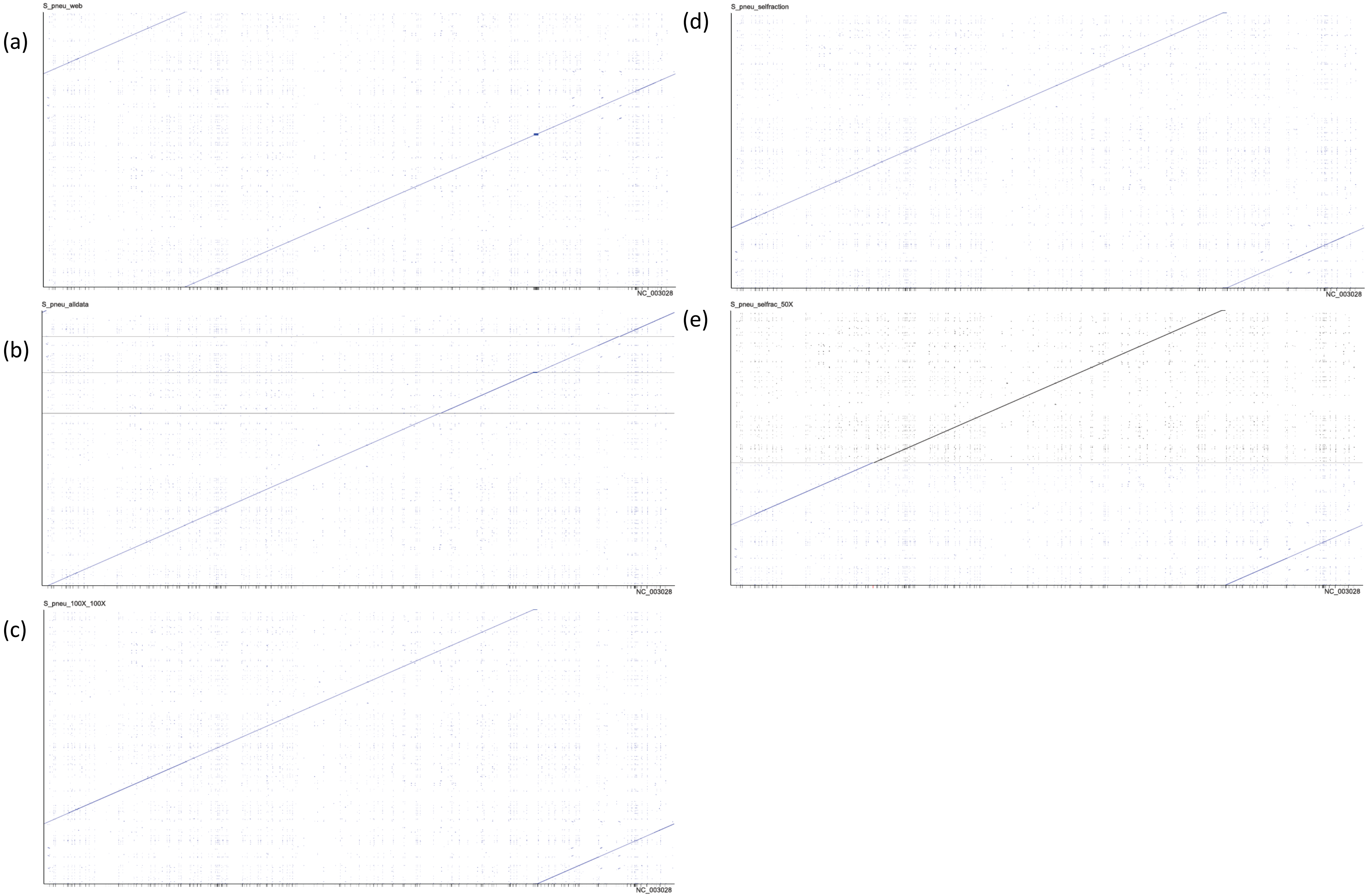

Figure S3 Dot plots of sequences assemblies produced by ALLPATHS-LG on dataset 3. (a) Website data, (b) Raw data, (c) 100X coverage, (d) Fractional data, and (e) 50X coverage.

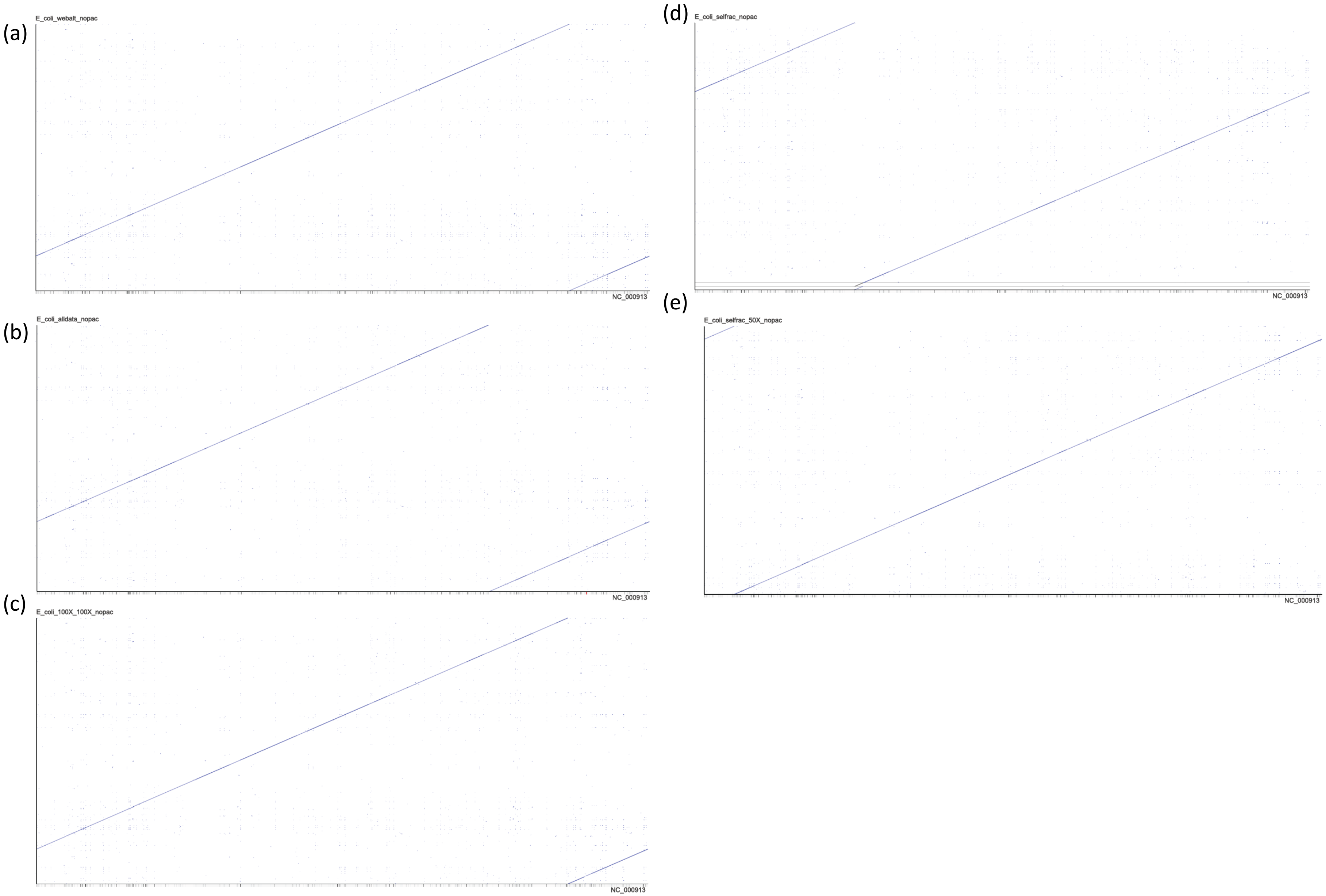

Figure S4 Dot plots of sequences assemblies produced by ALLPATHS-LG on dataset 1 without PacBio long reads. (a) Website data, (b) Raw data, (c) 100X coverage, (d) Fractional data, and (e) 50X coverage.

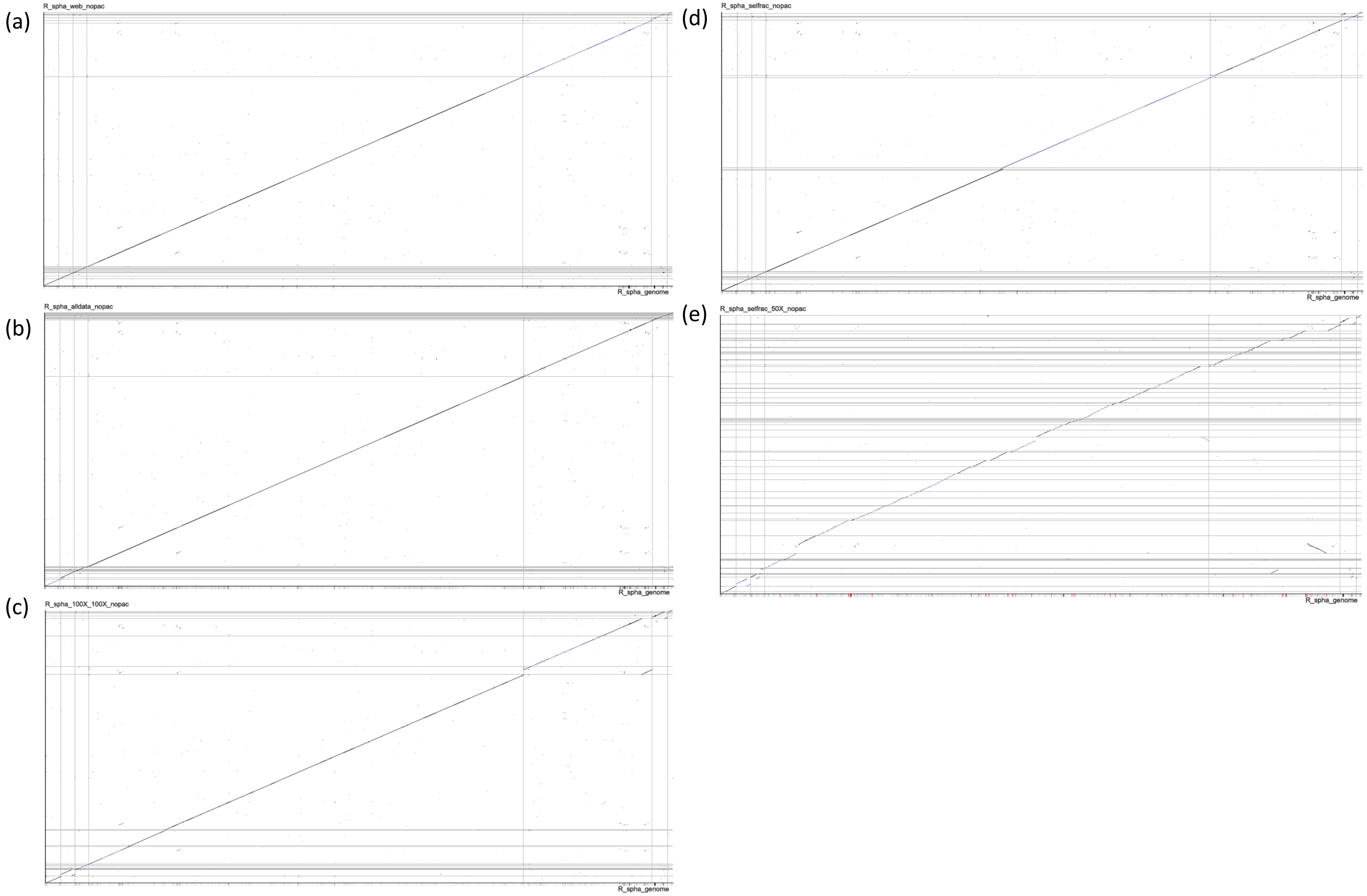

Figure S5 Dot plots of sequences assemblies produced by ALLPATHS-LG on dataset 2 without PacBio long reads. (a) Website data, (b) Raw data, (c) 100X coverage, (d) Fractional data, and (e) 50X coverage.

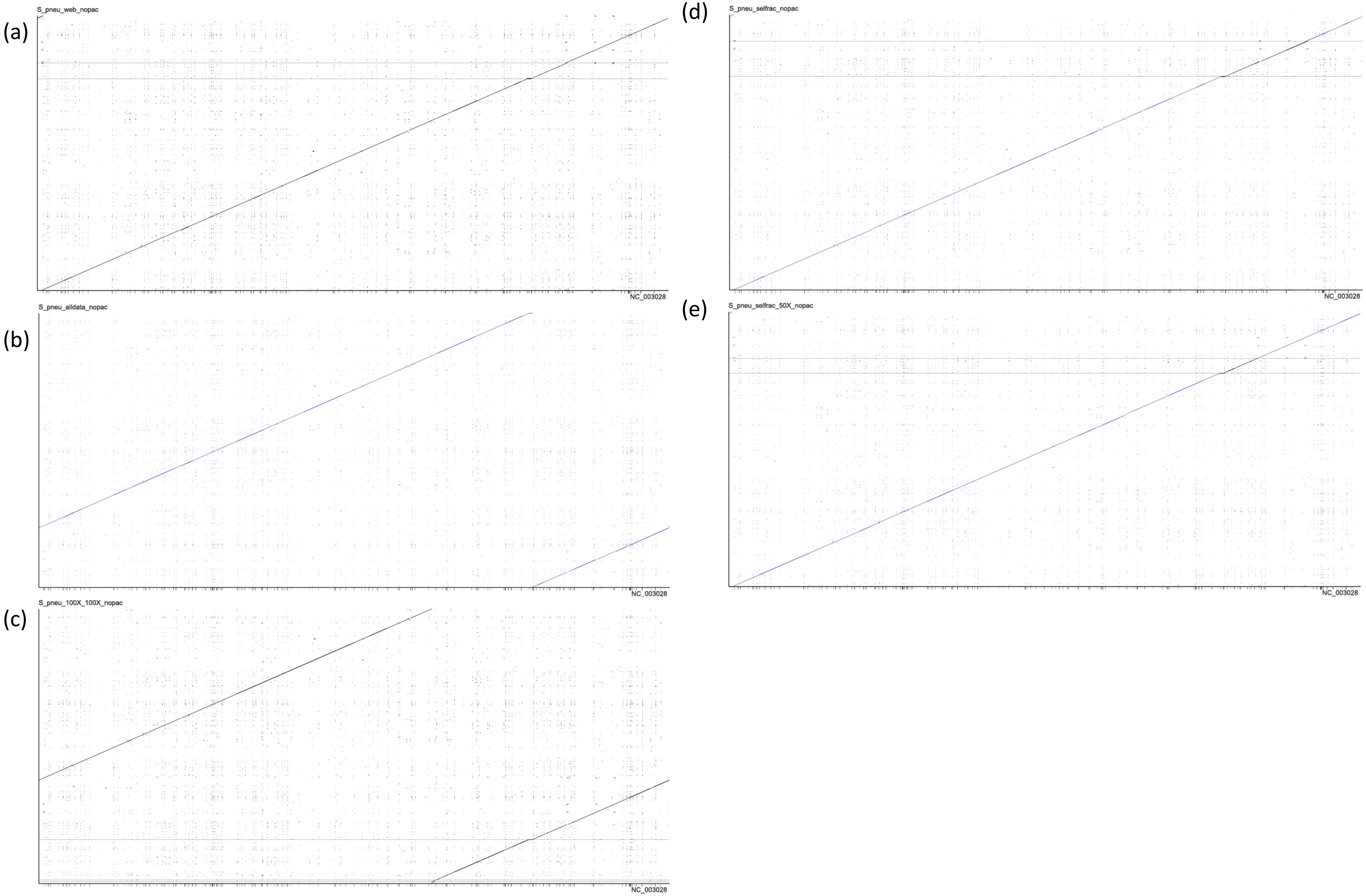

Figure S6 Dot plots of sequences assemblies produced by ALLPATHS-LG on dataset 3 without PacBio long reads. (a) Website data, (b) Raw data, (c) 100X coverage, (d) Fractional data, and (e) 50X coverage.

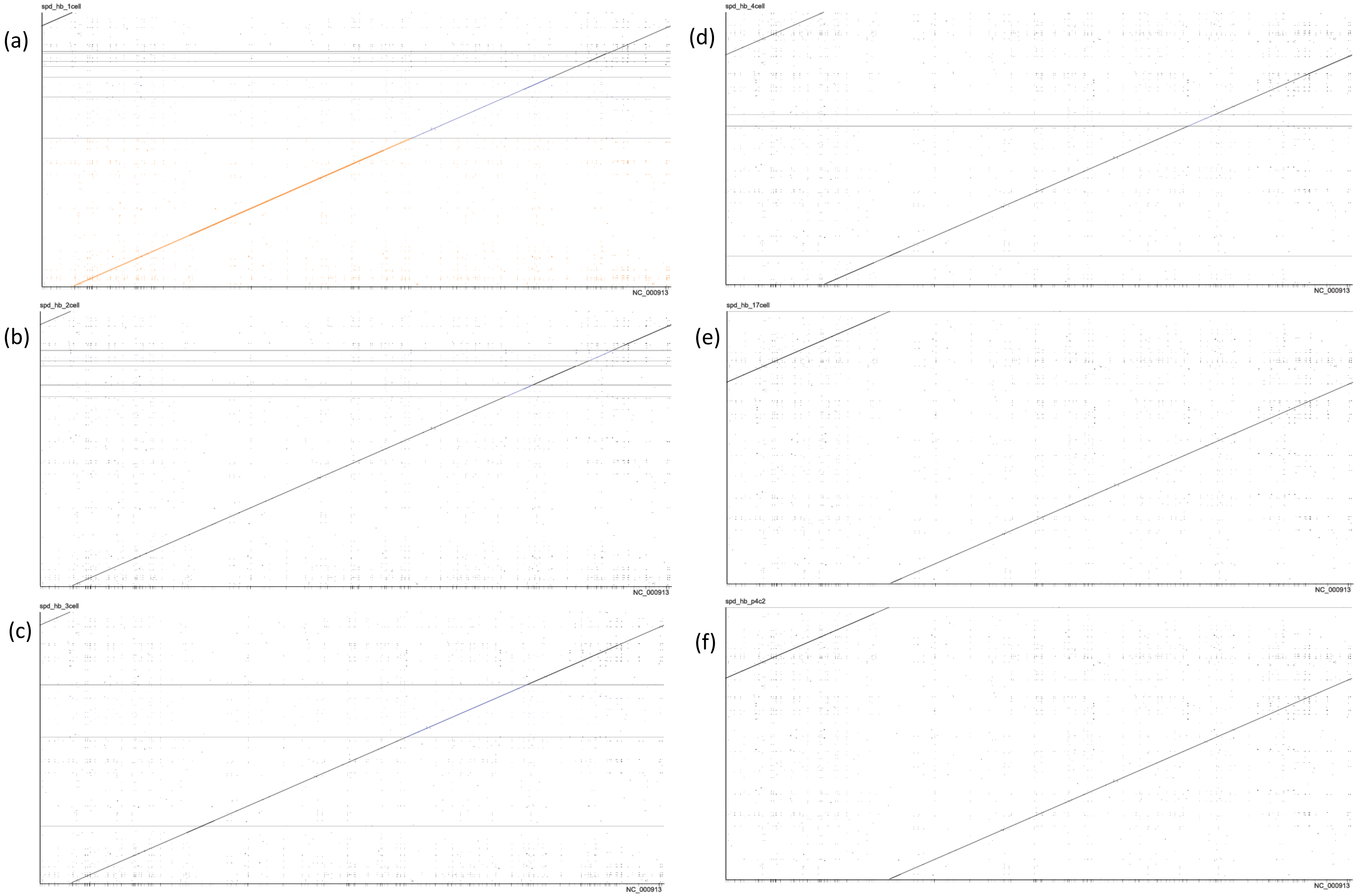

Figure S7 Dot plots of sequences assemblies produced by SPAdes on the hybrid datasets. The dataset of short reads is D4. The long reads are from (a) 1 SMRT cell,(b) 2,(c) 3,(d) 4,(e) 17 SMRT cells of dataset D5, and (f) 1 SMRT cell of dataset D9.

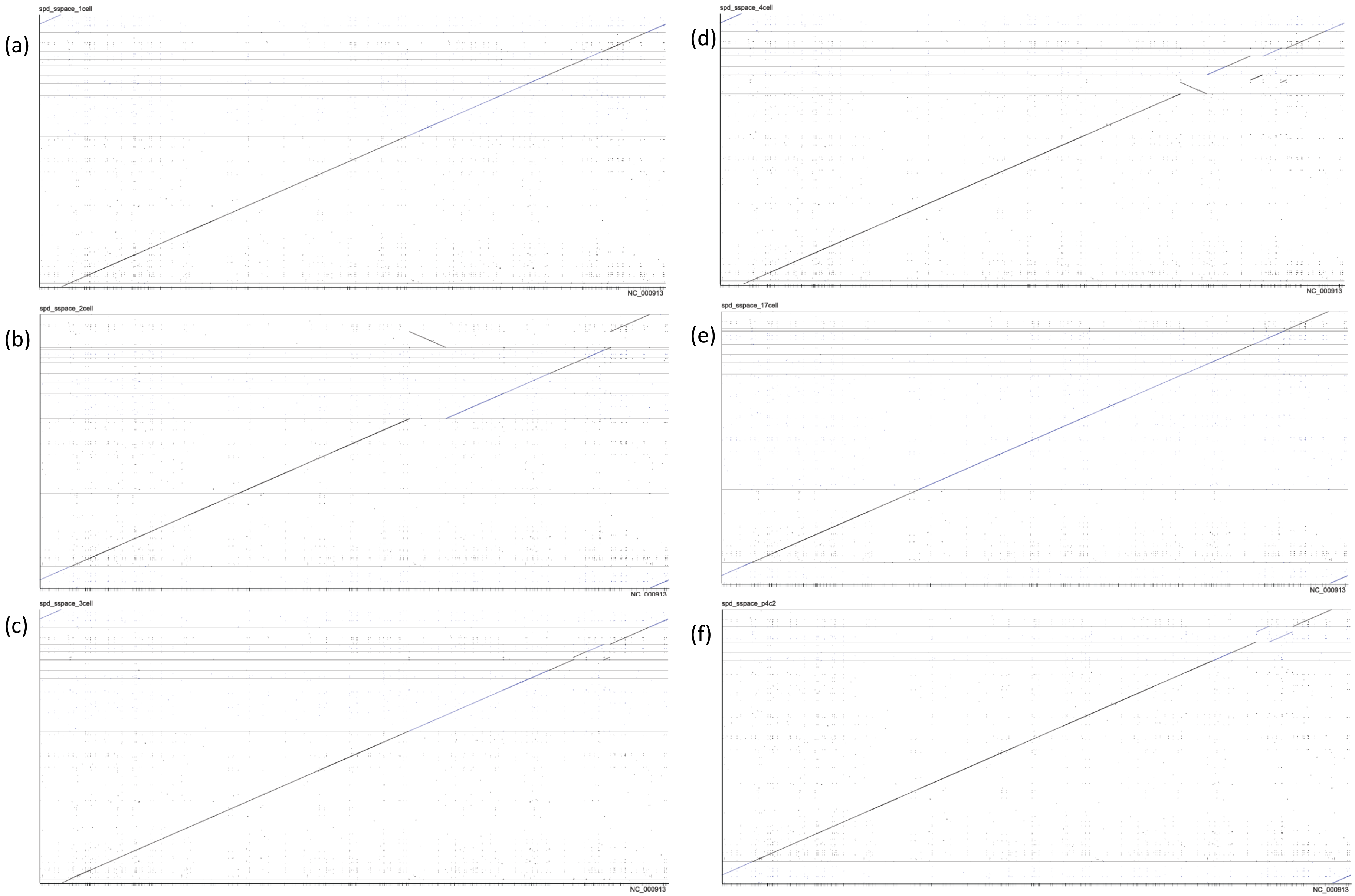

Figure S8 Dot plots of sequences assemblies produced by SSPACE-LongRead for scaffolding the assembly constructed by SPAdes using dataset D4. The long reads are from (a) 1 SMRT cell,(b) 2,(c) 3,(d) 4,(e) 17 SMRT cells of dataset D5, and (f) 1 SMRT cell of dataset D9.

(a) (b)

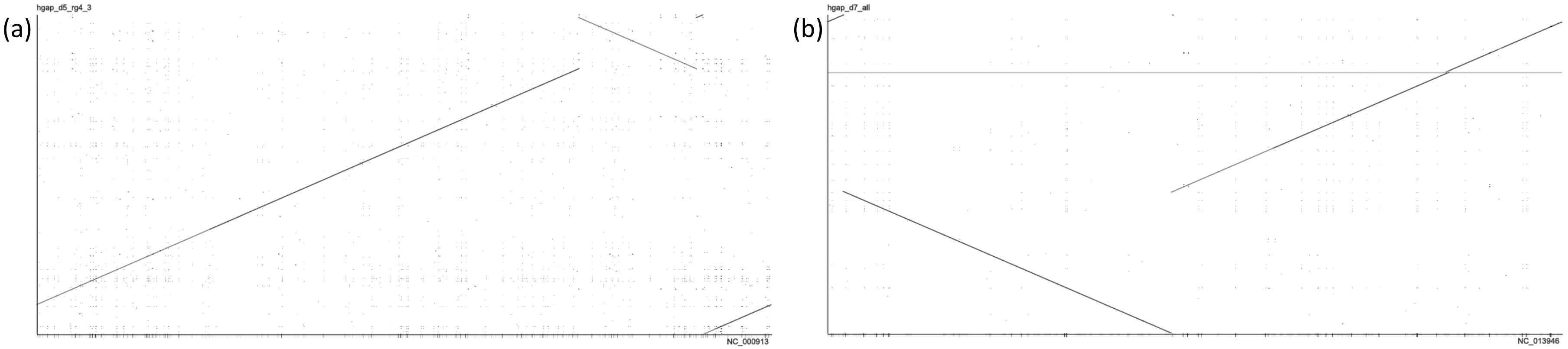

Figure S9 Dot plots of sequences assemblies produced by HGAP. (a) 4 SMRT cells of the dataset D5: the 3rd set, and (b) 4 SMRT cells of the dataset D7

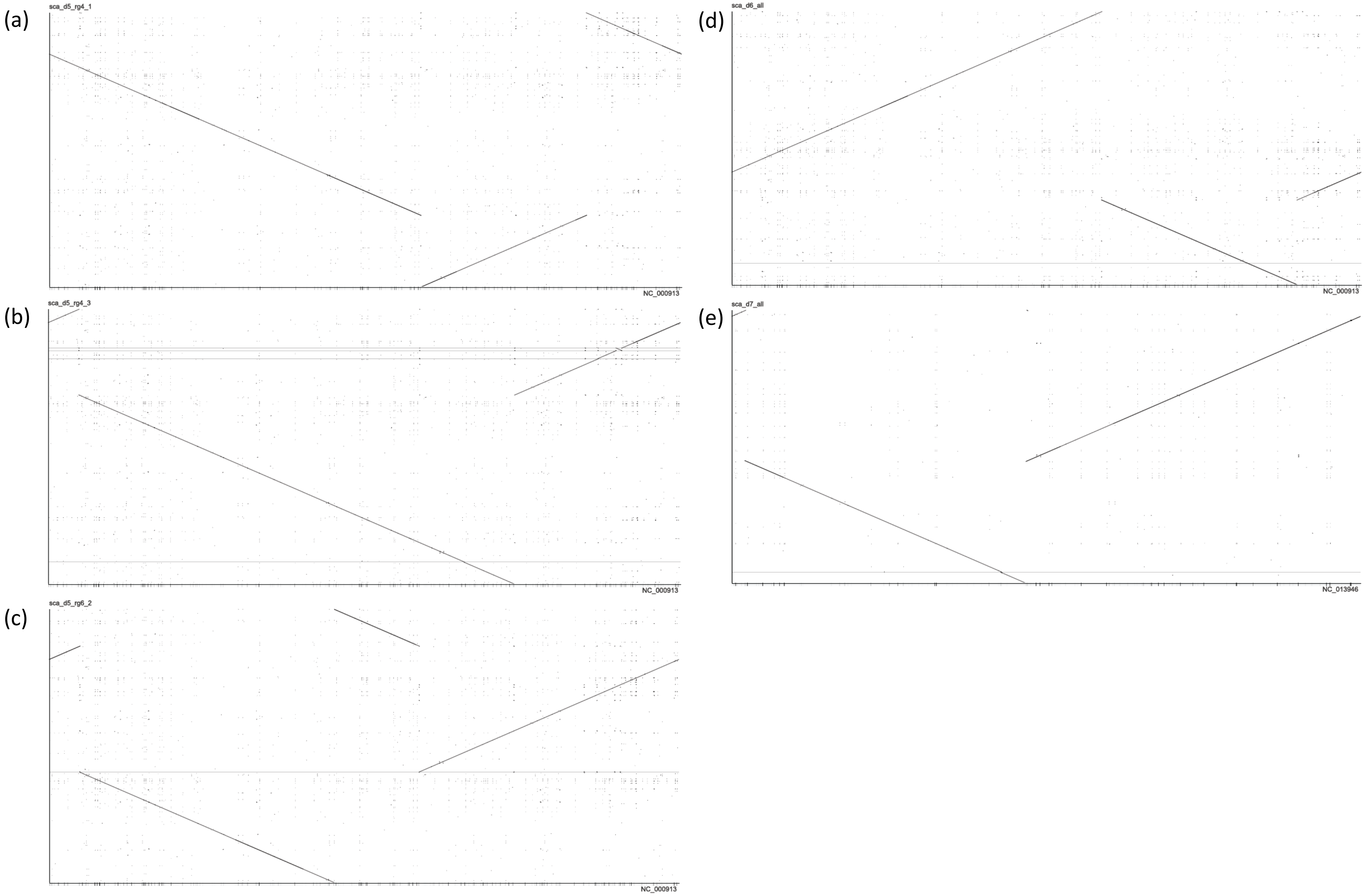

Figure S10 Dot plots of sequences assemblies produced by PBcR pipeline(S). (a) 4 SMRT cells: the 1st set, (b) 4 SMRT cells: the 3rd set, (c) 6 SMRT cells: the 2nd set of the dataset D5, (d) 8 SMRT cells of the dataset D6, (e) and 4 SMRT cells of the dataset D7.

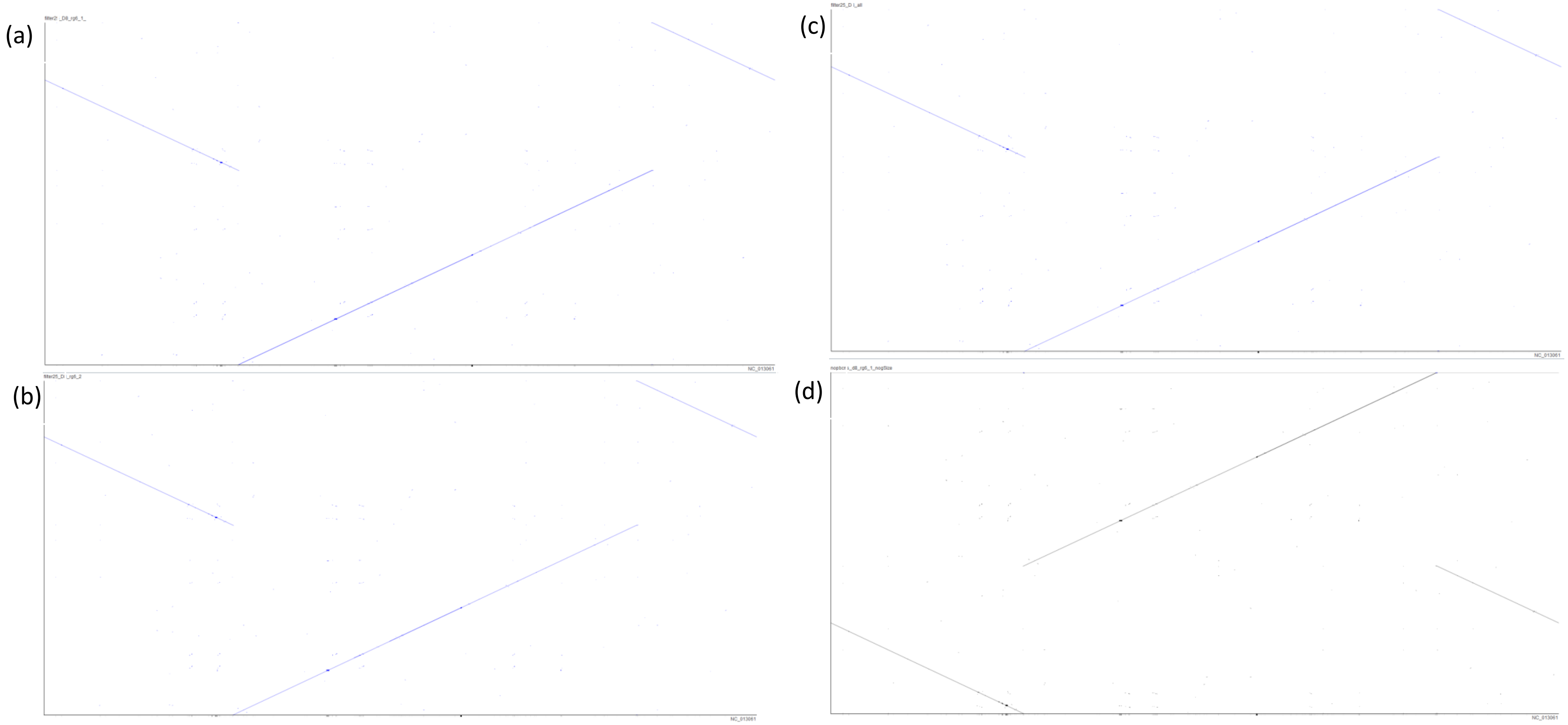

Figure S11 Dot plots of sequences assemblies produced by the latest PBcR pipeline(S) in wgs-8.2 without genome size setting. (a) 6 SMRT cells: the 1st set, (b) 6 SMRT cells: the 2nd set, (c) 7 SMRT cells of the dataset D8, (d) and 6 SMRT cells: the 1st set without specifying "-pbCNS" in PBcR

Supporting Data: Details descriptions about PBcR pipeline

In 2012, Koren *et al.* published a method to achieve hybrid *de novo* assembly of bacterial genomes [1]. The authors utilized high-accuracy short reads to correct errors in long reads and then assembled the corrected long reads, such an approach was called PBcR pipeline [2]. Similarly, a hybrid concept was leveraged by Bashir *et al.* in finishing bacterial genomes [3]. Later, Koren *et al.* successfully reduced the assembly complexity of microbial genome using the PBcR pipeline with only PacBio long reads by self-correction, abbreviated to "PBcR pipeline(S)" [4]. As the authors provided the compiled source code and sequencing data, they have generated short reads produced by Illumina MiSeq and long reads from 17 SMRT cells for *E. coli* (D4 and D5 in Table 1); we thus evaluated this hybrid approach (PBcR pipeline [2]) proposed by Koren *et al.* [1] on these combined data, i.e. dataset D4 and D5. In addition, to analyze the raw data with optional parameters, the pre-processed data (100X Miseq and 200X filtered subreads) was downloaded from the location provided by Koren *et al.* [4], for the sake of comparison. The pre-compiled algorithms executed under the hybrid approach were downloaded from http://www.cbcb.umd.edu/software/PBcR/closure/; these algorithms are identical to PBcR pipeline(S).

In order to execute the PBcR pipeline, both short- and long-read data are required. Short reads are employed in the correction of errors present in long reads at the

command of pacBioToCA. Subsequently, corrected long reads (PacBio corrected reads, PBcR) are assembled at the behest of runCA. Although the main two commands are adopted in the hybrid approach, there are several issues that must be considered. Firstly, the results of the assembly operation in terms of the number of contigs and the total length were substantially affected by the depth of short reads, long reads and PBcR, which can be seen in the left panel of Figure S12. The quantity of contigs was increased to 286 when the depths of the short and long reads were increased to the raw data (~373X) and 4 SMRT, respectively; the total length of this assembly was found to be exceptionally high (over 6 Mb, while the *E. coli* genome is roughly 4.6Mb in length). Following the recommendation described in the website (http://sourceforge.net/apps/mediawiki/wgs-assembler/index.php?title=PBcR), the approach was modified to obtain the longest 25X of PBcR prior to assembling the components. Harnessing 25X PBcR for assembly (Figure S12, in box) was found to reduce the number of contigs and maintain the total length of the assemblies. For example, the number of contigs was reduced from 123 to 23 and the longest contig among the assembly was increased from 0.69 Mb to over 1.2Mb when 100X short reads were employed to correct 4 SMRT-cell long reads and subsequently assemble the longest 25X PBcR. According to the method described by Koren *et al.*, an assembly was obtained by discarding contigs with fewer than 100 mapped PBcR [4].

We therefore excluded the contigs containing less than 100 reads (Figure S12, right panel). As can be observed in Figure S12, excluding the contigs with less confidence indeed rids the assembly of redundant contigs, *i.e.* the total length of assemblies approximates the genome size, and the number of contigs reduced to a reasonable range between 12 and 40. Nevertheless, the hybrid assembly that was obtained from the raw short reads, in addition to the long reads of the 4 SMRT cells, was not complete owing to the fact that the number of contigs was 12 and the longest contig was as small as 1.8Mb. Secondly, in executing pacBioToCA, the amount of PBcR was influenced by the genomeSize parameter, especially when the coverage of long read was high (>50X). For example, the seventy-fold filtered subreads of 4 SMRT cells was corrected by means of pacBioToCA without and with genomeSize=4650000 setting, along with 118X short reads, to generate 58X and 42X PBcR, respectively (as shown in Table S10). However, in similarity to the assembly obtained from the concatenation of the raw short reads with the 4 SMRT-cell long reads, the optimal genome was assembled from the combination of the 118X short reads with the 4 SMRT-cell long reads into 10 contigs; the longest contig, 2.0 Mbp, was still incomplete. Thirdly, runCA, the Celera Assembler executive script [5], was utilized to reconstruct a genome from PBcR. It is recommended to execute Celera Assembler with optional parameters. Two different settings for runCA were hence evaluated to

assemble the PBcR obtained from hybrid correction using the 200X long reads and the 100X short reads (data released from [4]). The QUAST-evaluated assemblies are summarized in Table S11. Although Koren *et al.* has assembled the genome to as few as 2 contigs, the present approach was unable to reproduce the assembly using the identical datasets under the two different settings.

Table S10 Hybrid assemblies obtained from combining 4 SMRT cells (70X) with 118X short reads; correction was performed using pacBioToCA with/without genome size setting

	Without genome size		With genome size	
Depth of PBcR	58.3X	25X	42.3X	25X
Average length	2252.7	5185.2	3495.6	5371.8
No. of contig	21	26	14	10
N50	412226	285200	544950	904105
Longest contig	983533	621920	1099298	2034167
Total length	4654299	4508804	4703352	4678847
No. of misassemblies	15	9	14	10
No. of N's per 100 Kbp	0.04	0	0	0
Genome fraction (%)	99.687	97.023	99.931	99.961
No. of genes	4474+15	4344+37	4489+6	4491+4

Table S11 Hybrid assemblies using runCA employing different parameters, PBcR were obtained using pacBioToCA with genome size on the data combining 200X long reads (from 17 SMRT cells) with 100X short reads

Depth of PBcR	40.1X	25X	40.1X	25X
Average length	4927.7	7278.7	4927.7	7278.7
Parameter	runCA1[a]		runCA2[b]	
No. of contig	7	5	16	7
N50	1478089	1215597	678445	1253429
Longest contig	2069213	2021284	1204169	1754178
Total length	4635310	4666475	4744173	4690899
No. of misassemblies	10	8	8	6
No. of N's per 100 kbp	0	0.02	0.08	0
Genome fraction (%)	99.561	99.944	99.85	100
No. of genes	4467+12	4487+6	4491+6	4492+5

[a] The spec file (asm.spec) describing the recommended parameters for assembly was downloaded from http://sourceforge.net/apps/mediawiki/wgs-assembler/index.php?title=PacBioToCA

[b] The parameters were recommended to make overlap detection more stringent by specifying the following parameters "unitigger=bogart merSize=14 ovlMinLen=<ovl value> utgErrorRate=0.015 utgGraphErrorRate=0.015 utgGraphErrorLimit=0 utgMergeErrorRate=0.03 utgMergeErrorLimit=0" in runCA, where the <ovl value> was set to approximately 40% of the average length of the corrected sequences.

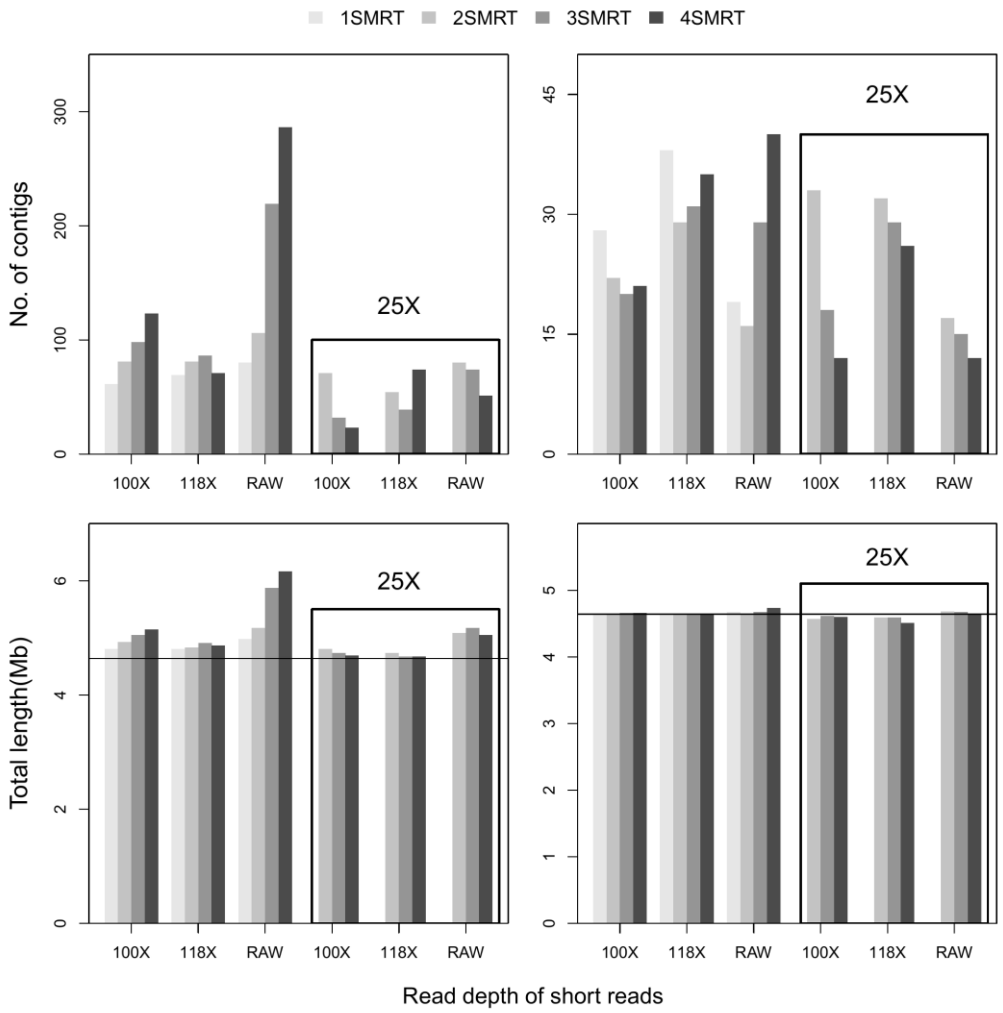

Figure S12 Effect of read depth on hybrid assemblies. PacBio long reads from one to four SMRT cells were reconciled by means of 100X, 118X, and raw (373X) Illumina short reads to produce PacBio corrected reads (PBcR). The PBcR and the longest 25X of the PBcR (in box) were assembled with Celera Assembler. The number of contigs and the total number of base pairs for each assembly are summarized (left panel). Contigs with fewer than 100 reads were discared to produce the final assemblies (right panel).

References

1 Koren, S. *et al.* Hybrid error correction and de novo assembly of single-molecule sequencing reads. *Nature biotechnology*, doi:10.1038/nbt.2280 (2012).

2 Utturkar, S. M. *et al.* Evaluation and validation of de novo and hybrid assembly techniques to derive high quality genome sequences. *Bioinformatics*, doi:10.1093/bioinformatics/btu391 (2014).

3 Bashir, A. *et al.* A hybrid approach for the automated finishing of bacterial genomes. *Nature biotechnology*, doi:10.1038/nbt.2288 (2012).

4 Koren, S. *et al.* Reducing assembly complexity of microbial genomes with single-molecule sequencing. *Genome Biol* **14**, R101, doi:10.1186/gb-2013-14-9-r101 (2013).

5 Myers, E. W. A Whole-Genome Assembly of Drosophila. *Science* **287**, 2196-2204, doi:10.1126/science.287.5461.2196 (2000).
